# Reserves, Injury Severity, and Outcomes in Traumatic Brain Injury: A CENTER‐TBI Observational Study

**DOI:** 10.1002/acn3.70509

**Published:** 2026-08-09

**Authors:** Natascha Ekdahl, Marika C. Möller, Lindsay Wilson, Anne Vik, Toril Skandsen, Jonas Stenberg, CENTER‐TBI participants and investigators

**Affiliations:** ^1^ Department of Clinical Sciences Danderyd Hospital, Karolinska Institutet Stockholm Sweden; ^2^ Center for Research and Development Uppsala University/County Council of Gävleborg Gävle Sweden; ^3^ Faculty of Health and Occupational Studies University of Gavle Gävle Sweden; ^4^ Department of Rehabilitation Medicine Danderyd University Hospital Stockholm Sweden; ^5^ Division of Psychology, Faculty of Natural Sciences University of Stirling Stirling UK; ^6^ Department of Neuromedicine and Movement Science Norwegian University of Science and Technology (NTNU) Trondheim Norway; ^7^ Department of Neurosurgery St. Olav's University Hospital Trondheim Norway; ^8^ Clinic of Rehabilitation St. Olav's University Hospital Trondheim Norway

**Keywords:** cognitive reserve, traumatic brain injury, injury severity

## Abstract

**Objective:**

Reserve refers to the brain's ability to maintain function after an injury and strongly relates to traumatic brain injury (TBI) outcomes. This study examined (1) whether associations between pre‐injury reserve proxies and outcomes differed across injury severity categories, and (2) whether the impact of injury severity varied across functional, symptomatic, and cognitive outcomes.

**Methods:**

This observational cohort study used data from CENTER‐TBI. Of 4509 enrolled patients, 3377 were between 18 and 75 years and alive 6 months post‐injury. Outcomes included functional outcome (GOSE, *n* = 2790), self‐reported symptoms (RPQ, *n* = 1470), and cognition (composite score, *n* = 1968). Pre‐injury reserve proxies were education, age, and psychiatric history. Injury severity was categorized as uncomplicated mild TBI, complicated mild TBI, moderate, and severe TBI.

**Results:**

Of nine tested interactions between reserve proxies and injury severity, only the interaction of age with injury severity on cognition was significant (*β* = 0.1, *p* < 0.001), with age being less influential in severe TBI. Higher education and no psychiatric history predicted better outcomes across all severities. Younger age predicted more favorable GOSE and cognitive scores. RPQ symptoms peaked among adults aged 46–60. Increasing injury severity was associated with progressively lower odds of GOSE ≥ 6. RPQ scores plateaued in moderate TBI. Cognitive performance was similar in uncomplicated and complicated mild TBI.

**Interpretation:**

Pre‐injury reserve proxies predicted outcome largely independent of injury severity, with higher reserves linked to better recovery. Injury severity showed a graded relationship with functional outcome. Symptom burden and cognitive impairment demonstrated non‐linear patterns across severity categories.

**Trial Registration:**

ClinicalTrials.gov identifier: NCT0221022

Reserve refers to the ability of the brain to maintain function despite disease or injury and is known to be an important influence on traumatic brain injury (TBI) outcomes. Of the reserve concepts, cognitive reserve is the most established, defined as differences in the adaptability of cognitive processes in response to brain pathology [1]. It is typically estimated using proxies, like education or occupation. Cognitive reserve has proven useful in the context of neurodegenerative diseases and is also gaining relevance in TBI [2, 3, 4, 5, 6, 7, 8].

Other forms of reserve thought to influence brain recovery are brain reserve and psychological resilience, the latter also referred to as emotional reserve [1, 4, 9, 10]. Brain reserve is commonly estimated through structural brain measures like overall volume or cortical thickness [1].

Since aging is associated with brain atrophy, age is sometimes used as a coarse and clinically feasible proxy for brain reserve. However, it is an imprecise measure that neither captures underlying structural capacity nor reflects the non‐linear nature of brain atrophy across the lifespan [3, 11, 12]. Emotional reserve refers to the ability to maintain mental health under stress and is commonly assessed through self‐report questionnaires [13, 14]. Emotional reserve is closely related to psychiatric well‐being and a history of psychiatric illness has also been associated with post‐concussion symptoms and poorer functional outcome following mild TBI (mTBI) [15, 16].

Although age, education, and psychiatric history are well‐established predictors of outcome after TBI and have been examined within the CENTER‐TBI dataset, prior studies have typically assessed these factors within single outcome domains, restricted age or severity groups, and without considering potential interactions [17, 18, 19, 20]. Consequently, it remains unclear whether these pre‐injury reserve proxies interact with injury severity or exhibit differential associations across functional, symptomatic, and cognitive outcomes. The large CENTER‐TBI cohort spanning the full spectrum of injury severity offers an opportunity to address this issue.

TBI is conventionally classified as mild, moderate, or severe in emergency settings, with mild TBI often further divided into uncomplicated and complicated based on CT findings. A key question is whether reserve offers equal protection at different levels of injury severity. One hypothesis is that higher reserve no longer confers advantages in severe brain pathology [21]. This study primarily aimed to examine whether the effect of proxy measures of reserve, namely education, age and previous psychiatric history, varied by injury severity category. A secondary aim was to examine whether the effect of injury severity categories on outcome varied by outcome type.

## 1 Methods

### 1.1 Study Design

Patients were selected from CENTER‐TBI, a multicentre observational cohort study recruiting patients with TBI between December 19, 2014, and December 17, 2017. In total, 65 centres from 18 countries participated. Patient inclusion criteria were a diagnosis of TBI, an indication for a computed tomography (CT) scan and presentation to a medical centre within 24 h of injury. Exclusion criteria were a pre‐existing neurological disorder severe enough to interfere with outcome assessments. In the current study the 6‐month outcome assessments were used. Ethics approval was obtained from each recruiting site and consent for participation was obtained from all patients or their proxies. Further details can be found in previous CENTER‐TBI publications [22, 23].

### 1.2 Participants and Procedure

**FIGURE 1:**
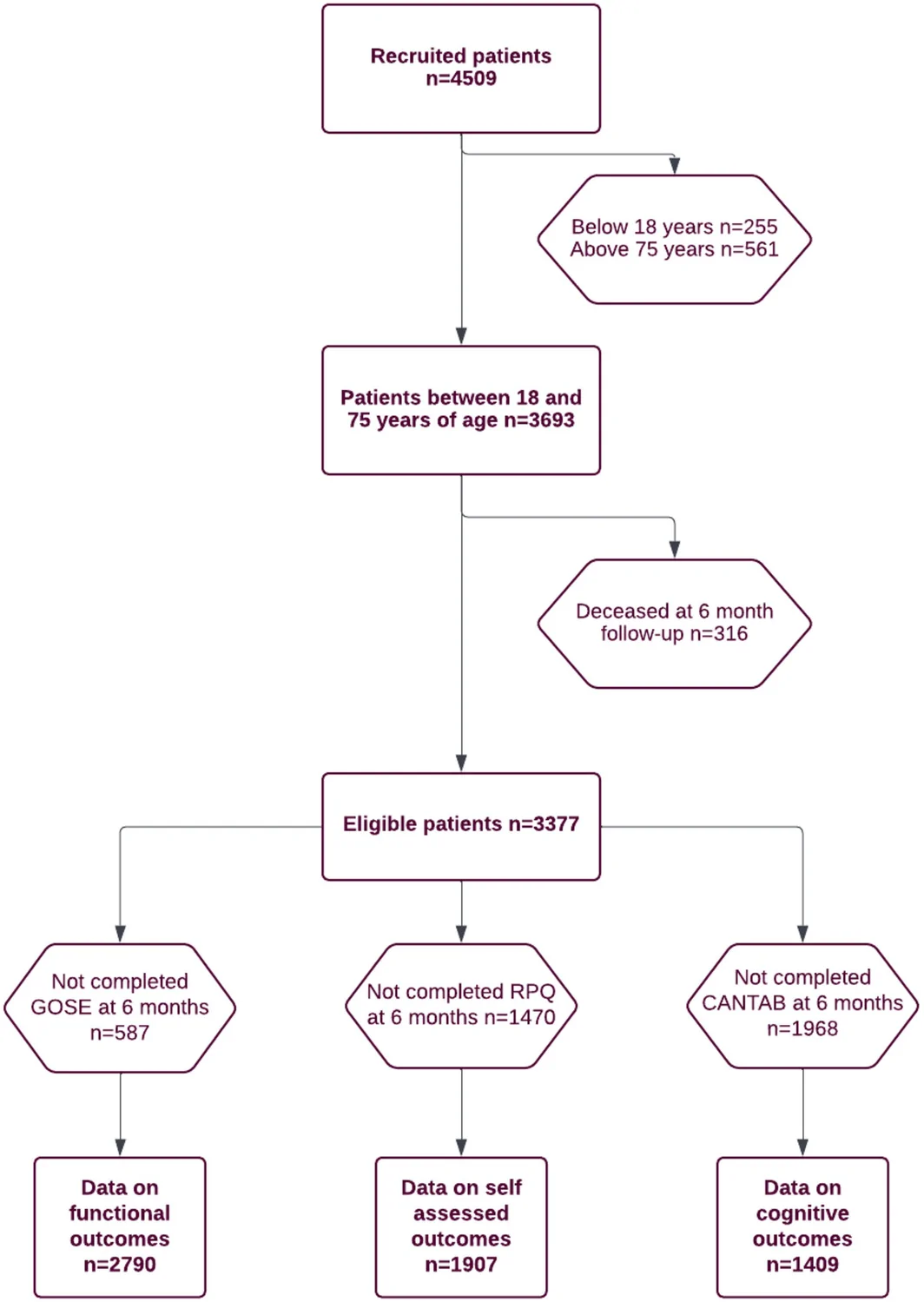
Flowchart of the participants included in the present study.

In the CENTER‐TBI core study 4509 participants were enrolled. In the current study, patients between 18 and 75, still alive at the 6‐month follow‐up were included; in total 3377 participants. Among these, 2790 had scores on Glasgow Outcome Scale—Extended (GOSE [24]) at 6 months after injury, 1907 had answered the Rivermead Post‐Concussion Symptoms Questionnaire (RPQ [25]) and 1409 had completed a cognitive assessment with the Cambridge Neuropsychological Test Automated Battery (CANTAB [26]) and traditional pencil and paper tests (see Figure 1).

Demographic and injury‐related data were collected at baseline. All patients were scheduled for follow‐up at 6 months. Patient completing the neuropsychological assessment were seen in person, while GOSE or RPQ could also be completed via phone or post. Further project details can be found in Steyerberg et al. [22].

### 1.3 Measures of Injury Severity

Injury severity was classified using the Glasgow Coma Scale (GCS) and initial CT findings (for the mild TBI) into four categories: Uncomplicated mTBI (mTBICT‐, GCS score 13–15, normal CT), complicated mTBI (mTBICT+, GCS score 13–15, intracranial abnormality on CT), moderate TBI (GCS score 9–12), and severe TBI (sTBI, GCS score 3–8). The Abbreviated Injury Scale (AIS) was used to rate the non‐brain injuries, ranging from 1 (minor) to 6 (fatal).

### 1.4 Measures of Cognitive, Brain and Emotional Reserve

*Cognitive reserve* was proxied using years of education, cross‐checked against the highest education level. Implausible values were corrected using median values for each education level (see Table S1). Education was capped at 21 years, reflecting the typical duration to attain a doctoral degree.

*Brain reserve* was proxied by age. Participants above 75 years were excluded due to a greater likelihood of other, age‐related diseases, confounding results.

*Emotional reserve* was proxied using psychiatric history, recorded by investigators and coded as either ‘yes’ or ‘no’ based on documented prior psychiatric diagnoses in the patients' medical records at recruitment.

### 1.5 Outcome Measures

#### 1.5.1 Functional Outcome—GOSE

The GOSE assesses functional outcome after brain injury, ranging from 1 (dead) to 8 (full recovery) [27]. While often dichotomized at 4/5, we used a cut‐off of ≥ 6 to define favorable recovery, as this typically reflects partial return to work. In CENTER‐TBI, the GOSE score reflects overall functioning without separating brain injury from other injuries. Alternative cut‐offs were tested as sensitivity analyses (see Tables S5 and S6).

#### 1.5.2 Self‐Reported Symptoms—RPQ

The RPQ assesses 16 cognitive, emotional, and somatic post‐concussion symptoms on a 5‐point scale. Scores range through 0 (never experienced at all), 1 (no more of a problem), 2 (mild problem), 3 (moderate problem) and 4 (severe new problem), with a total score of 0–64.

#### 1.5.3 Composite Cognitive Score

The composite cognitive score followed Wilson et al. [28] and included Rey Auditory Verbal Learning Test (RAVLT [29]) total recall, the Trail Making Test (TMT [29]) A and B completion times, and six CANTAB subtests covering memory, processing speed, attention, and executive function. Raw scores were standardized (mean = 0, SD = 1) using data from the uncomplicated mTBI category. A minimum of 6 out of 12 scores was required for a composite score to be calculated. Testing followed the study manual, with trained assessors. Participants scoring < 65 on the Galveston Orientation and Amnesia Test were excluded from cognitive testing.

### 1.6 Statistical Analysis

Statistical analysis was performed in R Studio version 4.4.1 [30]. Missing data were handled using multiple imputation by chained equations, using the mice‐package in R, under a missing at random assumption. Imputations were performed separately for each outcome domain (functional, self‐reported, and cognitive) to account for differences in analytic samples, covariate structures, and missingness patterns. Each imputation model included all variables in the corresponding analysis model along with additional variables associated with missingness. As results were consistent across datasets, only imputed results are reported; descriptive statistics for both original and imputed data are in the supplement (see Tables S2–S4).

All models included education, age, psychiatric history, region (North/West vs. South/East as used previously by CENTER TBI researchers [31]) and sex as main effects. Interactions between injury severity and each reserve factor (education, age, psychiatric history) were tested separately for each outcome domain (GOSE, RPQ, and cognitive scores). Interaction significance was assessed using Type III Wald tests. Non‐significant interactions were excluded when estimating main effects, while models with significant interactions retained these terms in subsequent analyses.

GOSE scores were analyzed using binary logistic regression (cut‐off ≥ 6), as the proportional odds assumption for ordinal regression was violated. Although the Box‐Tidwell test suggested non‐linearity for age, visual inspection showed minimal deviation, and age was therefore retained as a linear predictor.

RPQ scores were analyzed using negative binomial regression due to score distribution. Including 1‐scores improved model fit without altering significance patterns. Despite underestimating zero counts, a zero‐inflated model was not used, as the focus was on symptom intensity. Age was treated as a categorical variable (18–45, 46–60, 61–75) due to a non‐linear relationship with RPQ scores, with a peak observed in the 46–60 group based on visual inspection of plotted data. Coefficients are presented as incidence rate ratios (IRRs). An IRR greater than 1 indicates an increase in the expected event rate associated with a one‐unit increase in the predictor, whereas an IRR less than 1 indicates a decrease.

Cognitive scores were analyzed using linear regression. Assumptions of linearity, homoscedasticity and absence of outliers (Cook's distance) were met. All regression models were also checked for multicollinearity, which was low (VIF < 2).

Because not all patients completed every outcome measure, analyses were conducted on different samples. As a sensitivity check, the regression analyses for GOSE and RPQ were repeated in the cognitive composite subgroup (i.e., participants with complete data on all outcomes). Also, to assess potential selection bias due to non‐random missingness in cognitive outcomes, we conducted a sensitivity analysis under a missing‐not‐at‐random assumption. A proportion of missing cognitive scores was replaced with randomly generated values centered two standard deviations below the sample mean, representing a conservative scenario in which patients unable to complete testing had substantially poorer cognitive performance. This procedure was repeated in increments of 10% of missing cases.

## 2 Results

Descriptive characteristics of participants are presented in Table 1.

**TABLE 1:** Descriptive characteristics of participants.

Characteristic^a^	Uncomplicated mild TBI, *n* = 1214	Complicated mild TBI, *n* = 986	Moderate TBI, *n* = 412	Severe TBI, *n* = 644	*p* ^b^
Years of education, *m* (SD)	14.1 (4.0)	13.6 (3.9)	13.4 (3.8)	12.1 (3.4)	< 0.001
Unknown	85 (7%)	152 (15%)	101 (25%)	162 (25%)	
Age^c^, *m* (SD)	44.6 (16.7)	49.2 (16.8)	47.4 (16.7)	42.1 (16.5)	< 0.001
Psychiatric history					< 0.001
No, *n* (%)	1031 (85%)	842 (86%)	337 (82%)	510 (80%)	
Yes *n* (%)	174 (14%)	129 (13%)	63 (15%)	94 (15%)	
Unknown *n* (%)	9 (0.7%)	15 (1.5%)	12 (2.9%)	40 (6.2%)	
Sex					< 0.001
Female, *n* (%)	422 (35%)	309 (31%)	130 (32%)	156 (24%)	
Patient strata					< 0.001
ER, *n* (%)	598 (49%)	94 (9.5%)	6 (1.5%)	0 (0%)	
Admission, *n* (%)	519 (43%)	516 (52%)	91 (22%)	6 (0.9%)	
ICU, *n* (%)	97 (8.0%)	376 (38%)	315 (76%)	638 (99%)	
Length of stay, median (IQR)	1 (0, 2)	5 (2, 11)	12 (6, 25)	25 (14, 43)	< 0.001
Unknown, *n* (%)	8 (0.7%)	31 (3%)	15 (4%)	26 (4%)	
AIS score ≥ 3 (injuries requiring hospital admission), for other regions than head, *n* (%)	517 (43%)	838 (85%)	383 (93%)	633 (98%)	< 0.001
GOSE score at 6 months					< 0.001
2 or 3, *n* (%)	17 (1.4%)	32 (3.2%)	38 (9.2%)	155 (24%)	
4, *n* (%)	22 (1.8%)	30 (3.0%)	29 (7.0%)	55 (8.5%)	
5, *n* (%)	39 (3.2%)	81 (8.2%)	71 (17%)	109 (17%)	
6, *n* (%)	78 (6.4%)	136 (14%)	50 (12%)	85 (13%)	
7, *n* (%)	224 (18%)	210 (21%)	57 (14%)	70 (11%)	
8, *n* (%)	619 (51%)	339 (34%)	93 (23%)	60 (9.3%)	
Unknown, *n* (%)	215 (18%)	158 (16%)	74 (18%)	110 (17%)	
Rivermead Post‐Concussion Score (including 1), median (IQR)	8 (2, 19)	12 (4, 21)	13 (6, 24)	14 (7, 22)	< 0.001
Unknown, *n* (%)	533 (44%)	394 (40%)	181 (44%)	336 (52%)	
Cognitive composite score, median (IQR)	−0.06 (−0.54, 0.41)	−0.19 (−0.68, 0.25)	−0.24 (−0.86, 0.23)	−0.44 (−0.94, 0.05)	< 0.001
Unknown, *n* (%)	684 (56%)	539 (55%)	258 (63%)	432 (67%)	

Abbreviations: AIS, Abbreviated Injury Scale; ER, Emergency Room; GOSE, The Glasgow Outcome Scale—Extended; ICU, intensive care unit; TBI, traumatic brain injury.

^a^ 121 of the 3377 patients did not have a Glasgow Coma Scale score and were not included in the table.

^b^ Differences between injury severity categories were tested using ANOVA, Kruskal–Wallis and *χ* ^2^‐test depending on variable level of measurement.

^c^ Minimum age 18 years and maximum age 75 years across all severities.

### 2.1 The Effect of Education, Age and Psychiatric History Across Injury Severity Categories

All reported models included education, age, psychiatric history, injury severity, sex, and region as explanatory variables.

#### 2.1.1 Functional Outcomes According to GOSE

**FIGURE 2:**
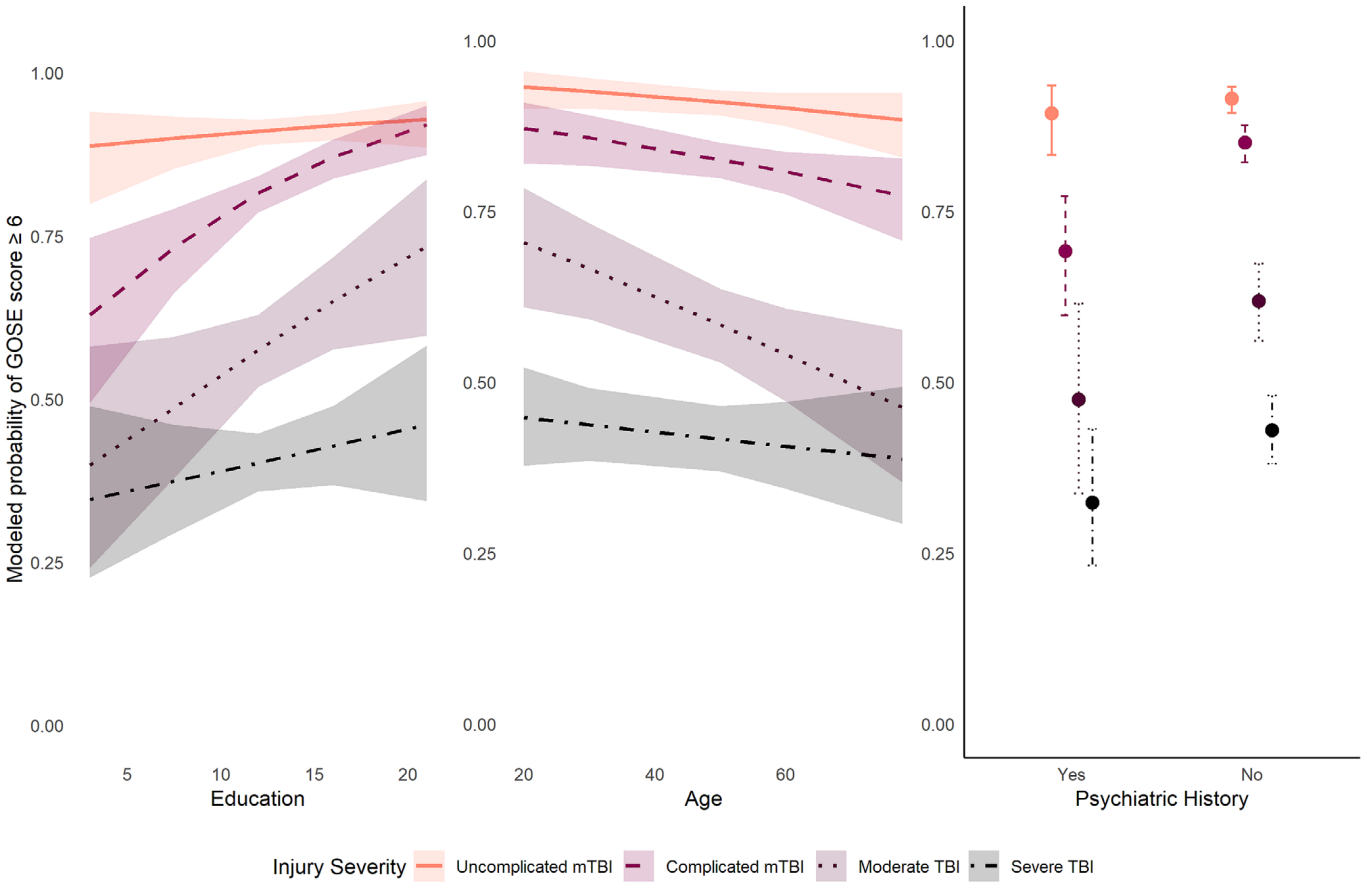
Effects of education, age, and psychiatric history on functional outcome according to GOSE, cut‐off value between 5 and 6. Region and sex were also included in the models. Shaded area represents 95% confidence intervals.

There were no significant interaction effects between any of the reserve proxies and injury severity for the GOSE (education by injury severity: *χ* ^2^(3) = 5.47, *p* = 0.14; age by injury severity: *χ* ^2^(3) = 2.46, *p* = 0.48; psychiatric history by injury severity: *χ* ^2^(3) = 3.82, *p* = 0.28) (Figure 2).

For illustrative purposes, Figure 2 shows the model with the (non‐significant) interaction terms. In the subsequent analysis, the non‐significant interaction term was removed, and only main effects were evaluated. Results from this reduced model are presented in Figure 5A. When the interaction term was omitted, education, age and psychiatric history all had a significant association with GOSE scores. Lower education, higher age and presence of previous psychiatric history were all associated with a lower likelihood of GOSE score ≥ 6. Alternative cut‐off levels were also tested (GOSE ≥ 5 and GOSE ≥ 7, respectively). No interactions between injury severity and reserve proxies were detected. However, psychiatric history was no longer significant in the GOSE ≥ 5 model, and age was no longer significant in the GOSE ≥ 7 model (see Tables S5 and S6).

#### 2.1.2 Self‐Reported Symptoms According to RPQ

**FIGURE 3:**
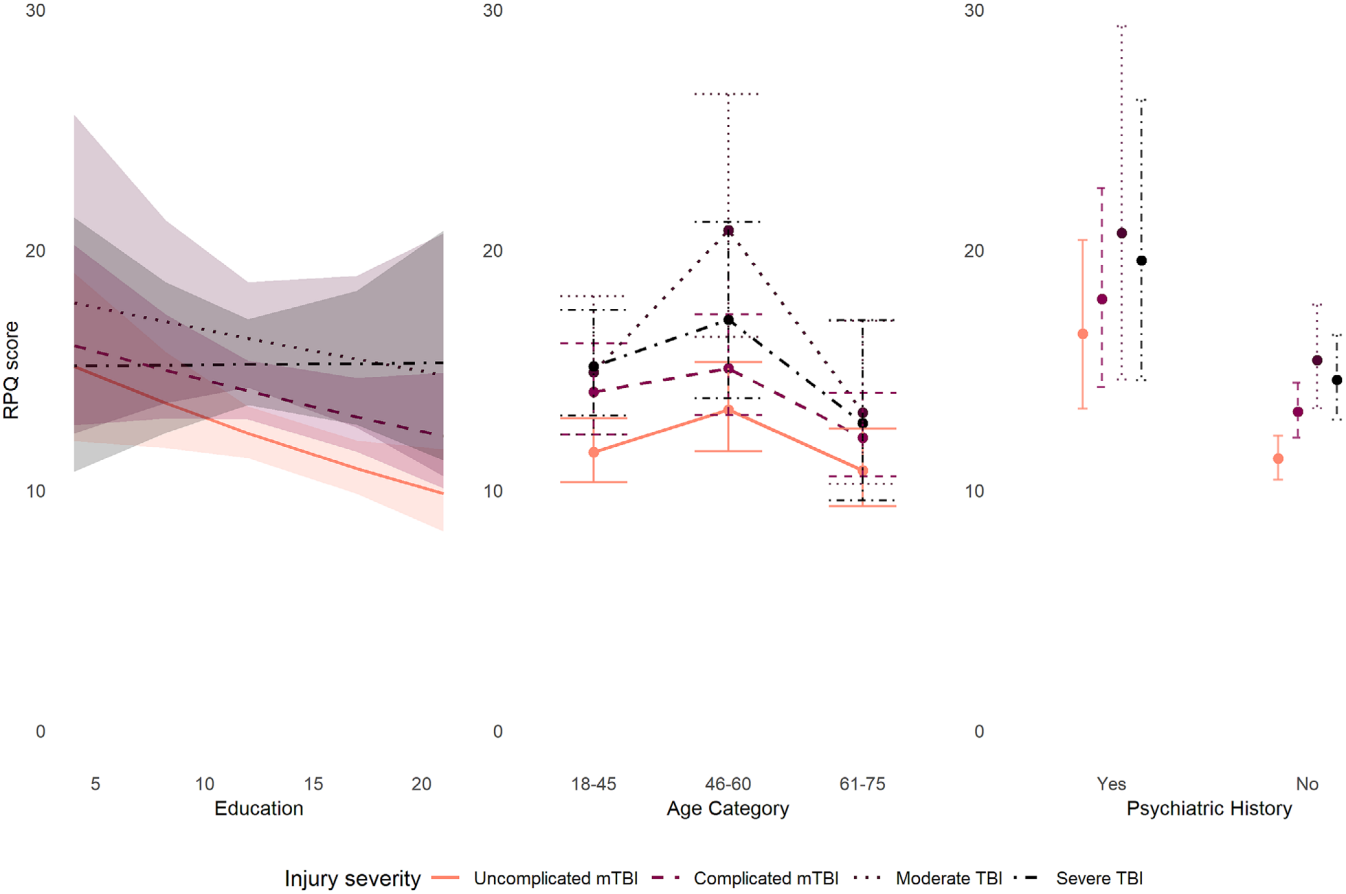
Effects of education, age, and psychiatric history on self‐reported symptoms (RPQ). Region and sex were also included in the models. Shaded area represents 95% confidence intervals.

There were no significant interactions between any of the reserve proxies and injury severity on self‐reported symptoms according to RPQ (education by injury severity: *χ* ^2^(3) = 1.62, *p* = 0.66; age by injury severity: *χ* ^2^(6) = 2.78, *p* = 0.84; psychiatric history by injury severity: *χ* ^2^(3) = 0.31, *p* = 0.99) (Figure 3).

Figure 3 represents the model with the non‐significant interaction terms. Since no interactions were significant, the model without interactions was used to estimate main effects. The model without interaction revealed significant main effects for education, age, psychiatric history and gender on self‐reported symptoms (Figure 5B). Higher education, no psychiatric history and male gender were associated with better outcomes. For age, the age span 46–60 was associated with more self‐reported symptoms.

#### 2.1.3 Cognition

**FIGURE 4:**
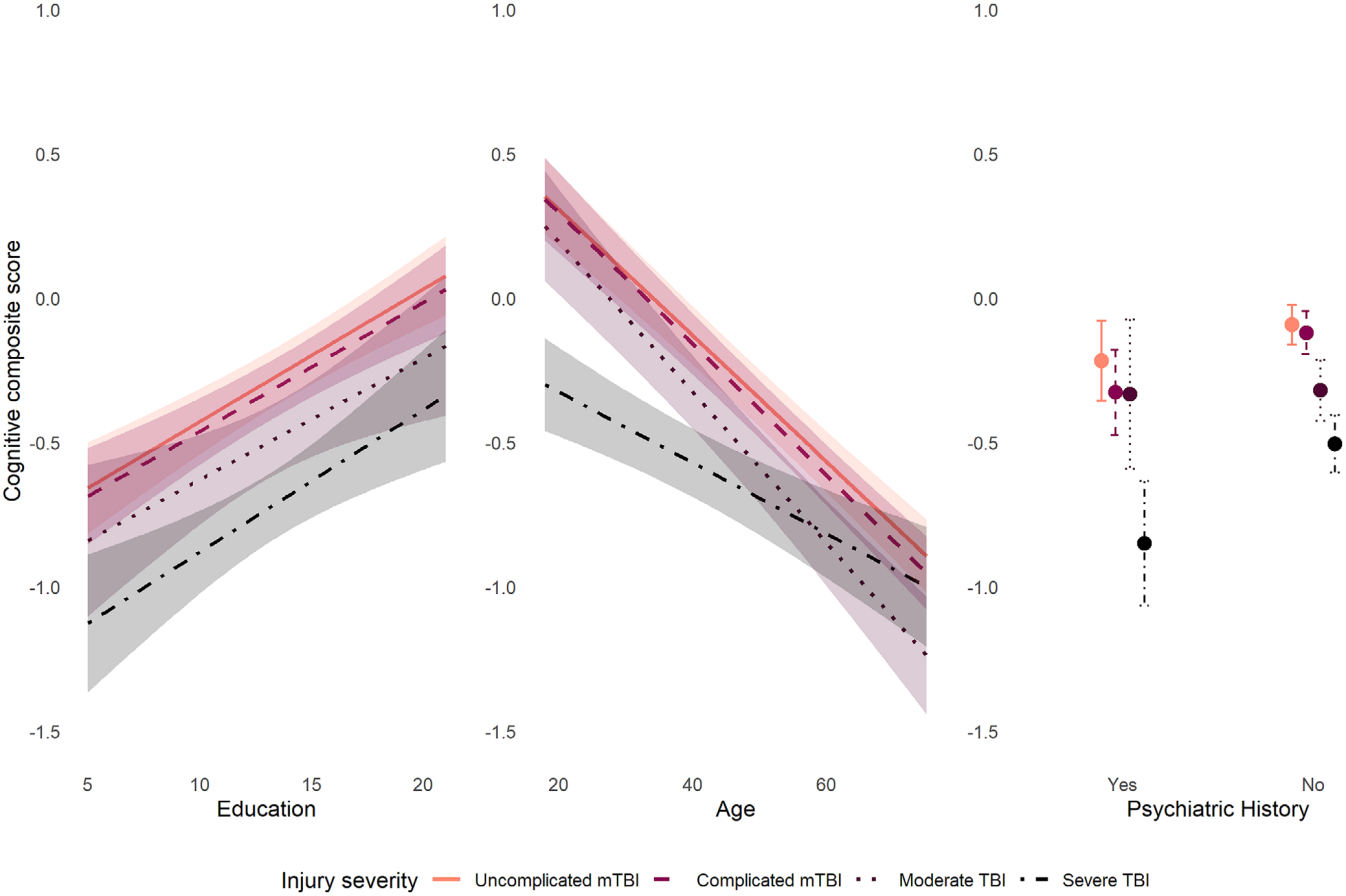
Effects of education, age, and psychiatric history on cognition. Region sex and were also included in the models. Shaded area represents 95% confidence intervals.

There was a significant interaction between age and injury severity on cognitive outcome (*F*(3, 1397) = 5.63, *p* = 0.00078). The slope of age in the most injured category was significantly less steep than in the other injury severity categories, indicating that the effect of age on cognitive scores was weakest in the most injured patients. Consequently, the effect of injury severity varied with age and the most pronounced differences in cognition between injury severity categories were seen in young patients (Figure 4). Only 30 participants (12%) with GOSE scores of 2 or 3 completed the cognitive assessment, compared to completion rates ranging from 43% to 67% among other GOSE levels. This suggests that a substantial portion of the missing data may be associated with lower cognitive functioning. To evaluate the robustness of the age and injury severity interaction under this assumption, sensitivity analyses were performed by imputing missing cognitive scores with low values, increasing the proportion imputed in 10% increments (e.g., 10%, 20%, 30%, etc.). When 20% of the missing cognitive scores were randomly replaced using a normal distribution centered two standard deviations below the observed mean, the Type III Wald test for the Age × Injury Severity interaction was still significant, *F*(3, 1633) = 2.72, *p* = 0.043. When 30% of the missing data were replaced, the Type III Wald test for the interaction was no longer significant, *F*(3, 1774) = 0.49, *p* = 0.69.

For the other two reserve proxies, there were no significant interactions with injury severity on cognitive scores (education vs. injury severity: *F*(3, 1397) = 0.054, *p* = 0.98; psychiatric history vs. injury severity: *F*(3, 1397) = 1.37, *p* = 0.25) (Figure 4).

**FIGURE 5:**
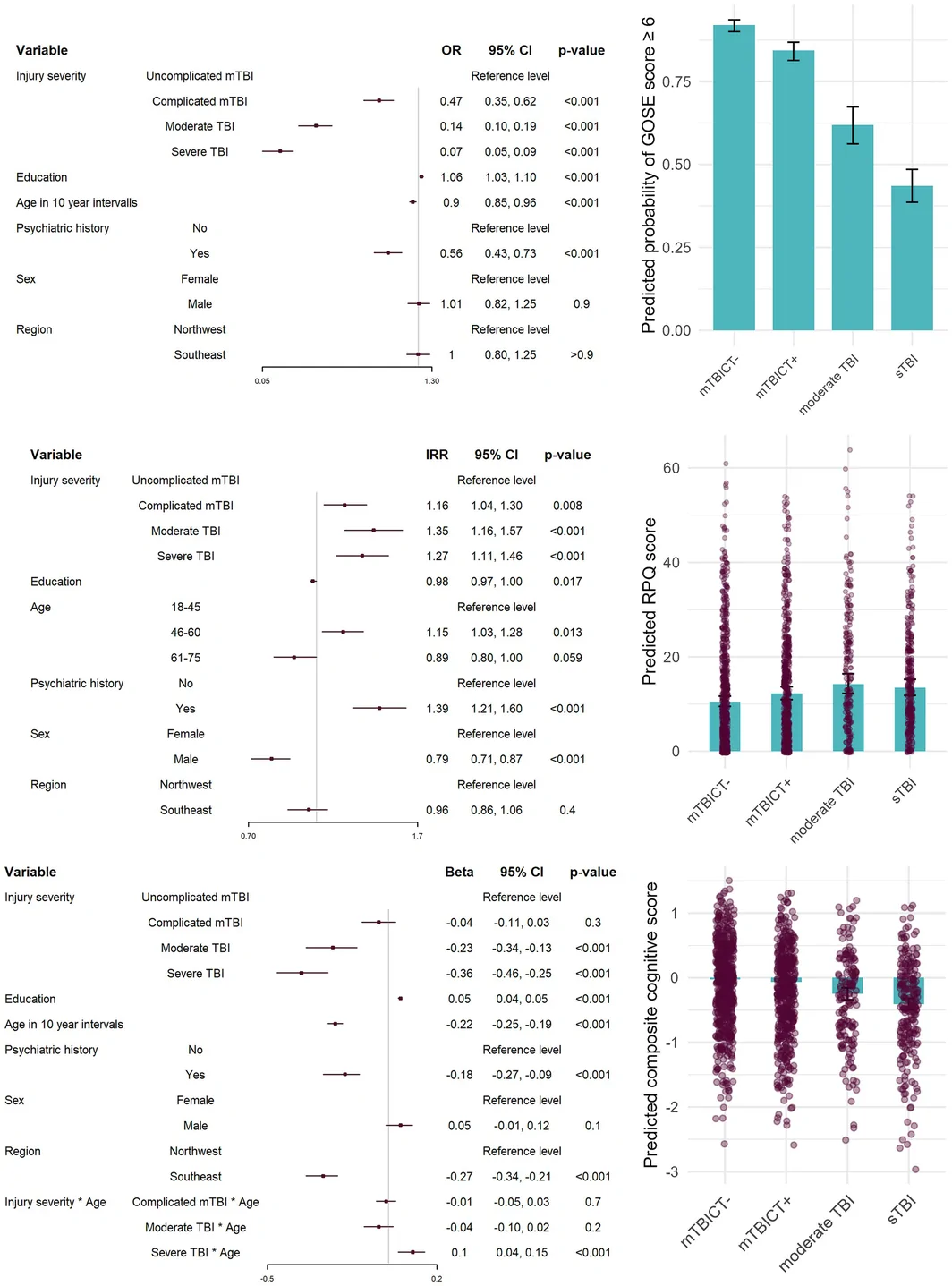
Forest plots and graphs illustrating the regression models examining the association between injury severity and outcome measures. Each model includes the three reserve variables (age, education, and psychiatric history), along with region and sex as covariates. CI, confidence interval; IRR, incidence rate ratio; mTBICT−, uncomplicated mild traumatic brain injury; mTBICT+, complicated mild traumatic brain injury; OR, odds ration; sTBI, severe traumatic brain injury. (A) Functional outcome, Glasgow Outcome Scale—Extended (GOSE), as the dependent variable, presented with main effects only (no interaction terms retained). (B) Self‐reported post‐concussion symptoms, measured with Rivermead Post‐Concussion Symptoms Questionnaire (RPQ), as the dependent variable, also presented with main effects only (no interaction terms retained). (C) shows the cognitive composite score as the dependent variable, retaining the significant interaction between age and injury severity categories. The reported betas for injury severity are at age = 49 (median age), and the beta for age is for uncomplicated mTBI.

Due to the significant interaction between age and injury severity, this model was retained for assessing main effects of the other factors. Lower education, higher age and psychiatric history were associated with lower cognitive scores (Figure 5C). Region also influenced scores, while sex did not.

#### 2.1.4 Sensitivity Check in the Sample With Complete Outcome Data

Since the different outcomes were completed by different subsamples of the total study population, the analyses of GOSE and RPQ were also conducted within the subsample that completed the cognitive measures, as this group had complete data for all outcomes. There were still no interactions between injury severity and reserve proxies. Results are presented in Tables S7 and S8.

### 2.2 Effect of Injury Severity Category Across Outcomes

In regression analyses, for functional outcome (GOSE score) every decrease in injury severity category corresponded to a higher probability of a GOSE score ≥ 6 (Figure 5A). Self‐reported symptoms (RPQ score) were also associated with severity, but moderate TBI showed the highest symptom scores, with only small differences between moderate and severe injuries (Figure 5B). For cognitive outcomes, the interaction effect between age and injury severity was retained for assessing the effect of injury severity categories on cognitive scores. Effects of the interaction with age were estimated at the sample median age to facilitate interpretation. At the median age (49 years), there was no difference in cognitive scores between the two mildest injury categories, but both moderate and severe injuries had significantly lower scores than milder injuries (Figure 5C).

## 3 Discussion

As already well established, education, age, and psychiatric history were found to be important for outcomes across all domains. A key finding in this study was that these factors were almost equally influential across all levels of injury severity. Although many previous studies have investigated the effect of reserve proxies on outcomes after TBI, potential interaction effects between injury severity and these proxies have rarely been studied. There is some previous evidence that age modifies recovery trajectory across levels of injury severity for functional outcomes, but education and psychiatric history have mainly been investigated as main effects, implicitly assuming that their impact is consistent across injury severity [32]. In the present study, only age showed a moderating effect on cognitive outcomes, with its impact on cognition being less pronounced in sTBI compared with the less severe injury categories. However, sensitivity analyses suggested that this could be due to selection bias. Consequently, this study supports the view that education, age, and psychiatric history are associated with functional, self‐reported and cognitive outcomes, but do not moderate the influence of injury severity on these outcomes, thereby suggesting that these variables do not provide post‐injury compensation but rather premorbid resilience.

Regarding the relationship between outcomes and injury severity, functional outcomes showed a consistent decline with increasing injury severity. Cognitive test scores were significantly lower in the moderate to severe injuries, whereas the two mildest injury categories showed only minimal differences in test performance. Self‐reported post‐concussion symptoms did not follow a linear pattern and patients with moderate and severe TBI reported a similar symptom load.

For functional outcomes, low education, higher age and previous psychiatric history all decreased the likelihood of scoring 6 or higher on the GOSE. As shown in the Supporting Information, varying the cut‐off only slightly altered the results. A cut‐off between 4 and 5 renders a history of psychiatric illness non‐significant, while a cut‐off between 6 and 7 renders age non‐significant. Education was significant in all instances. Regardless of cut‐off, there were no interactions between injury severity and the proxy variables of reserve on functional outcome.

Lower education and previous psychiatric history were significantly associated with higher ratings of self‐reported symptoms (RPQ). Age showed a non‐linear pattern, with individuals aged 46–60 reporting the most symptoms. There were no interaction effects between injury severity and reserve variables on RPQ scores. Patients with moderate TBI reported the highest RPQ scores after adjusting for demographics, though differences between moderate TBI and sTBI were minimal and not significant. This may reflect that the RPQ is designed to capture mTBI symptoms and not meant for use in sTBI, missing symptoms relevant for sTBI. Also, for RPQ scores, the graphical representation of data suggests that higher education is associated with greater RPQ scores in sTBI, consistent with the hypothesis that reserve may be less protective in more severe injuries [21]. This finding could also be due to selection bias, perhaps primarily participants with high education were well enough to complete the RPQ after sTBI, hence any true association between education and symptom severity in the sTBI group may have been obscured. Nevertheless, this relationship was not statistically significant. Females reported more symptoms than males, consistent with prior CENTER‐TBI findings [17].

Our results show that the effect of age on cognitive scores varies by injury severity, with age having less impact in sTBI. This could reflect the dominant role of the injury itself in sTBI, which may override the advantages associated with younger age. Age is also a predictor of mortality in sTBI, which may contribute to selection bias. Older individuals with the most severe injuries may not survive or at least not be able to complete a cognitive test, as indicated by the lower mean age in the sTBI category. In addition, the low rate of completed cognitive assessments among participants with low GOSE scores further supports the presence of selection bias, which may partly drive the observed interaction effect. Supporting this interpretation, our sensitivity analysis showed that when 30% of the missing cognitive outcome data were imputed as indicating severe cognitive impairment, the significant age × injury‐severity interaction was no longer present. The effects of education and psychiatric history were consistent across all severities, suggesting that the positive effect of higher cognitive and emotional reserve on cognitive function remain beneficial even in sTBI.

In line with previous research, education had a strong impact on cognitive performance. For example, individuals with high education and sTBI performed similarly to those with low education and mTBI. However, this finding applies only to patients who were able to complete the testing (i.e., the most severe cases were not included). No significant differences in cognitive performance were observed between individuals with uncomplicated and complicated mTBI. This supports previous concerns, such as those raised by Bigler et al. [33] that standard neuropsychological tests may lack the sensitivity to detect subtle deficits in mild TBI. It also suggests that cognitive impairments in mTBI, including complicated cases, probably are rare 6 months after injury [34, 35].

Most research on preinjury factors and reserve in TBI has focused on mTBI [8, 14, 36]. However, our findings suggest that the effects of variables related to reserve are largely consistent across injury severities. This aligns with previous studies on TBI as well as studies investigating the relationship between education and brain atrophy, linking higher education to greater regional cortical volume, but not slower rates of brain atrophy [7, 37]. These results imply that the beneficial effects of reserve stem from individuals possessing greater resources prior to injury, resources that persist post‐injury, rather than from reserve functioning as a compensatory mechanism. While some studies have reported a moderating effect of education on brain volume these cross‐sectional designs may reflect cohort effects or survivor bias rather than causality [38, 39].

Although conceptually distinct, all three reserve proxies showed similar associations with outcomes. This may suggest that education, age, and psychiatric history partly reflect different indicators of a broader latent domain related to premorbid capacity and resilience. Prior research supports this, linking higher education to both greater cortical volume and better psychological well‐being [37, 40]. However, the direction of these relationships remains unclear: does education enhance brain and emotional health, or do pre‐existing traits influence educational attainment? Longitudinal studies suggest early‐life cognitive ability better predicts later‐life cognition than education itself [41]. This implies that reserve may stem from underlying traits that drive both education and long‐term cognitive outcomes [42, 43].

### 3.1 Strengths and Limitations

This study's main strength is its large, multicentre European cohort spanning the full TBI severity spectrum. However, subgroup analyses were limited by smaller sample sizes, particularly in the moderate TBI and psychiatric history groups. Also, many variables had missing data, particularly in the sTBI category, which were addressed using multiple imputation. A potential limitation with the imputations was that they were performed separately for each outcome domain rather than within a single joint model, which may not fully preserve correlations between outcomes and could reduce efficiency; however, results were consistent with complete‐case analyses.

Another strength is the inclusion of multiple reserve and outcome measures, offering a broad perspective on reserve. Still, the reserve proxies have limitations. Education may vary across countries, mitigated here by controlling for region. Age and psychiatric history are crude indicators of brain and emotional reserve; more precise measures (e.g., cortical thickness, resilience scales) would be preferable. The relationship between age and brain atrophy is non‐linear and there is great variation in atrophy, especially at older ages [44, 45]. Psychiatric history was often based on medical records, potentially missing undiagnosed cases. It would have been preferable to include additional variables in the analysis to construct composite variables for the different forms of reserve. However, although CENTER‐TBI is an exceptionally rich dataset, it also exhibits the challenges typical of large multicenter studies, including inconsistencies in data collection that have resulted in substantial missingness for several variables. Therefore, we chose to focus on a smaller set of relatively complete variables that were collected consistently across sites. Outcome comparisons are constrained by format differences: GOSE is observer‐rated, RPQ is self‐reported, and CANTAB is performance‐based, introducing selection bias. Patients with severe injuries may be rated on GOSE but unable to complete RPQ or CANTAB. Nevertheless, sensitivity analyses showed consistent patterns in the subgroup with complete data. Another limitation is the use of a cut‐off for ‘good recovery’ on the GOSE, which naturally skews group proportions depending on the threshold. However, sensitivity analyses showed that varying the cut‐off did not lead to interaction effects between injury severity and functional outcomes. Finally, the severity classification used, mainly based on GCS, is a convenient categorization for research studies but not sufficiently nuanced when describing individual patients, hence not properly selecting patients for clinical trials or interventions [46]. Although new and more refined systems for classifying injury severity have recently been proposed, we elected to use the traditional categories. These classifications have been used in previous CENTER‐TBI publications and are familiar to clinicians, thereby making our results more readily comparable with earlier research and more easily applicable in clinical practice [46].

## 4 Conclusions

For each outcome studied, education, age and psychiatric history showed consistent effects across all severities, except for age, whose negative impact on cognition was lower in sTBI, likely due selection bias. Categories of injury severity were consistently associated with functional outcomes. For self‐reported symptoms and cognitive outcomes, increased injury severity was less consistently related to worse outcomes. Overall, these findings emphasize the importance of assessing variables related to reserve in clinical practice, as patients with lower educational attainment, older age, and a history of psychiatric conditions are more likely to experience poorer outcomes across all levels of TBI severity.

## Author Contributions

**Natascha Ekdahl:** methodology, data analysis, writing – original draft, writing – review and editing. **Marika C. Möller:** methodology, writing – original draft, writing – review and editing, supervision. **Lindsay Wilson:** conceptualization, methodology, writing – review and editing. **Anne Vik:** conceptualization, methodology, writing – review and editing. **Toril Skandsen:** conceptualization, methodology, writing – review and editing. **Jonas Stenberg:** conceptualization, methodology, resources, writing – original draft, writing – review and editing, supervision. Center‐TBI participants and investigators are listed in Appendix S1.

## Funding

Data used in preparation of this manuscript were obtained in the context of CENTER‐TBI, a large collaborative project with the support of the European Union 7th Framework Program (EC grant 602150). Additional funding was obtained from the Hannelore Kohl Stiftung (Germany), from OneMind (USA) and from Integra LifeSciences Corporation (USA).

## Disclosure

Methodology statement: Data for the CENTER‐TBI study has been collected through the Quesgen e‐CRF (Quesgen Systems Inc., USA), hosted on the INCF platform and LUMC Opal platform and extracted via the INCF LUMC Opal platform (LUMC, NL)*. Data were extracted from the CENTER‐TBI database 20th of October, 2023.

## Ethics Statement

The CENTER‐TBI study (EC grant 602150) has been conducted in accordance with all relevant laws of the EU if directly applicable or of direct effect and all relevant laws of the country where the recruiting sites were located, including but not limited to the relevant privacy and data protection laws and regulations (the “Privacy Law”), the relevant laws and regulations on the use of human materials, and all relevant guidance relating to clinical studies from time to time in force including, but not limited to, the ICH Harmonized Tripartite Guideline for Good Clinical Practice (CPMP/ICH/135/95) (“ICH GCP”) and the World Medical Association Declaration of Helsinki entitled “Ethical Principles for Medical Research Involving Human Subjects”. Informed Consent by the patients and/or the legal representative/next of kin was obtained, accordingly to the local legislations, for all patients recruited in the Core Dataset of CENTER‐TBI and documented in the e‐CRF. Ethical approval was obtained for each recruiting site. The list of sites, Ethical Committees, approval numbers and approval dates can be found on the website: https://www.center‐tbi.eu/project/ethical‐approval.

## Conflicts of Interest

The authors declare no conflicts of interest.

## Supporting information




**Table S1:** Cut‐off and replacement values for different levels of education.
**Table S2:** Imputed dataset Glasgow Outcome Scale—Extended (GOSE). Distribution of GOSE scores divided according to injury severity categories. TBI, traumatic brain injury.
**Table S3:** Imputed dataset Rivermead Post‐Concussion Symptoms Questionnaire (RPQ). Distribution of RPQ scores divided according to injury severity categories. IQR, interquartile range; TBI, traumatic brain injury.
**Table S4:** Imputed dataset Cognitive composite score. Distribution of cognitive composite scores divided according to injury severity categories. IQR, interquartile range; TBI, traumatic brain injury.
**Table S5:** Sensitivity test using GOSE cut‐off between 4 and 5. No statistically significant interactions were detected between any of the reserve variables and injury severity on likelihood of having a GOSE score of ≥ 5. The logistic regression models without the interaction effect found that psychiatric history was no longer significantly associated to outcome when the cut‐off for good recovery according to GOSE was chosen to be ≥ 5. GOSE, Glasgow Coma Scale Extended; TBI, traumatic brain injury.
**Table S6:** Sensitivity test using GOSE cut‐off between 6 and 7. No statistically significant interactions were detected between any of the reserve variables and injury severity on likelihood of having a GOSE score ≥ 7. The logistic regression models without the interaction effect found that age was no longer significantly associated with outcome when the cut‐off for good recovery according to GOSE was chosen to be ≥ 7. GOSE, Glasgow Coma Scale Extended; TBI, traumatic brain injury.
**Table S7:** Sensitivity test GOSE in the composite cognitive score sample. No significant interaction effects were found between injury severity and any of the reserve variables on Glasgow Outcome Scale‐Extended (GOSE) scores in the subset having complete cognitive scores.
**Table S8:** Sensitivity test RPQ in the composite cognitive score sample. No significant interaction effects were found between injury severity and any of the reserve variables on Rivermead Post‐Concussion Symptoms Questionnaire (RPQ) scores in the subset having complete cognitive scores.

**Appendix S1:** The CENTER‐TBI Participants and Investigators.

## Data Availability

The data that support the findings of this study are available from Center TBI. Restrictions apply to the availability of these data, which were used under license for this study. Data are available from https://www.centertbi.eu/data with the permission of Center TBI.
