# Supplementary material for: Reserves, Injury Severity, and Outcomes in Traumatic Brain Injury: A CENTER‐TBI Observational Study
Source: Ann Clin Transl Neurol. 2026 Aug 9:10.1002/acn3.70509. Online ahead of print. doi: 10.1002/acn3.70509 (PMC13454244; doi:10.1002/acn3.70509)
Supplement: Supplementary file 1 [file ACN3-9999-0-s001.docx]

## Supplemental tables

### Supplemental table 1: Cut-off and replacement values for different levels of education.

|  | Years of education | | |
| --- | --- | --- | --- |
| Education level | *Lower limit* | *Upper limit* | *Replacement value* |
| Primary school | 5 | 11 | 8 |
| Secondary school / High school | 8 | 16 | 12 |
| College / University (diploma or degree | 12 | - | 17 |
| Currently in diploma or degree-oriented program | 5 | - | 13 |
| None, not currently in school | - | 9 | 8 |
| Post-high school training (e.g. trade/technical certificate) | 8 | 18 | 14 |

### Supplemental table 2: Imputed dataset Glasgow Outcome Scale – Extended (GOSE)

| GOSE score at 6 months | Mild uncomplicated TBI n = 1024 | Mild complicated TBI n = 841 | Moderate TBI n = 347 | Severe TBI n = 529*^1^* |
| --- | --- | --- | --- | --- |
| 1 | 0 (0%) | 0 (0%) | 0 (0%) | 0 (0%) |
| 2 or 3 | 21 (2.1%) | 36 (4.3%) | 38 (11%) | 147 (28%) |
| 4 | 25 (2.4%) | 32 (3.8%) | 30 (8.6%) | 52 (9.8%) |
| 5 | 44 (4.3%) | 83 (9.9%) | 74 (21%) | 109 (21%) |
| 6 | 86 (8.4%) | 134 (16%) | 50 (14%) | 83 (16%) |
| 7 | 226 (22%) | 214 (25%) | 57 (16%) | 68 (13%) |
| 8 | 622 (61%) | 352 (41%) | 98 (28%) | 70 (13%) |

Distribution of GOSE scores divided according to injury severity categories. TBI: Traumatic brain injury

### Supplemental table 3: Imputed dataset Rivermead Post-Concussion Symptoms Questionnaire (RPQ)

|  | Mild uncomplicated TBI n = 709 | Mild complicated TBI n = 621 | Moderate TBI n = 235 | Severe TBI n = 324 |
| --- | --- | --- | --- | --- |
| RPQ score (including 1) Median (IQR); | 8 (2, 18) | 11 (4, 20) | 13 (6, 25) | 13 (7, 21) |

Distribution of RPQ scores divided according to injury severity categories. TBI: Traumatic brain injury; IQR: Interquartile range.

### Supplemental table 4: Imputed dataset Cognitive composite score

|  | Mild uncomplicated TBI n = 562 | Mild complicated TBI n = 466 | Moderate TBI n = 158 | Severe TBI n = 216 |
| --- | --- | --- | --- | --- |
| Cognitive composite score Median (IQR) | -0.07 (-0.54, 0.38) | -0.20 (-0.69, 0.24) | -0.25 (-0.83, 0.23) | -0.44 (-0.93, 0.04) |

Distribution of cognitive composite scores divided according to injury severity categories. TBI: Traumatic brain injury; IQR: Interquartile range.

### Supplemental table 5: Sensitivity test using GOSE cut-off between 4 and 5.

No statistically significant interactions were detected between any of the reserve variables and injury severity on likelihood of having a GOSE score of ≥ 5. The logistic regression models without the interaction effect found that psychiatric history was no longer significantly associated to outcome when the cut-off for good recovery according to GOSE was chosen to be ≥ 5. GOSE: Glasgow Coma Scale Extended; TBI: Traumatic Brain Injury.

### Supplemental table 6 Sensitivity test using GOSE cut-off between 6 and 7.

No statistically significant interactions were detected between any of the reserve variables and injury severity on likelihood of having a GOSE score >=7. The logistic regression models without the interaction effect found that age was no longer significantly associated with outcome when the cut-off for good recovery according to GOSE was chosen to be >=7. GOSE: Glasgow Coma Scale Extended; TBI: Traumatic Brain Injury

### Supplemental table 7 Sensitivity test GOSE in the composite cognitive score sample.

|  | Result | p-value |
| --- | --- | --- |
| Interaction severity x education | χ²(3)=2.51 | 0.47 |
| Interaction severity x age | χ²(3)=2.42 | 0.49 |
| Interaction severity x psychiatric history | χ²(3)=3.65 | 0.30 |

No significant interaction effects were found between injury severity and any of the reserve variables on Glasgow Outcome Scale-Extended (GOSE) scores in the subset having complete cognitive scores.

### Supplemental table 8 Sensitivity test RPQ in the composite cognitive score sample.

|  | Result | p-value |
| --- | --- | --- |
| Interaction severity x education | χ²(3)=0.09 | 0.99 |
| Interaction severity x age | χ²(3)=2.05 | 0.91 |
| Interaction severity x psychiatric history | χ²(3)=2.81 | 0.42 |

No significant interaction effects were found between injury severity and any of the reserve variables on Rivermead Post-Concussion Symptoms Questionnaire (RPQ) scores in the subset having complete cognitive scores.
