# Supplementary material for: Reserves, Injury Severity, and Outcomes in Traumatic Brain Injury: A CENTER‐TBI Observational Study
Source: Ann Clin Transl Neurol. 2026 Aug 9:10.1002/acn3.70509. Online ahead of print. doi: 10.1002/acn3.70509 (PMC13454244; doi:10.1002/acn3.70509)
Supplement: Supplementary file 2 [file ACN3-9999-0-s002.docx]

**The CENTER-TBI participants and investigators:**

Cecilia Åkerlund^1^, Krisztina Amrein^2^, Nada Andelic^3^, Lasse Andreassen^4^, Audny Anke^5^, Anna Antoni^6^, Gérard Audibert^7^, Philippe Azouvi^8^, Maria Luisa Azzolini^9^, Ronald Bartels^10^, Pál Barzó^11^, Romuald Beauvais^12^, Ronny Beer^13^, Bo-Michael Bellander^14^, Antonio Belli^15^, Habib Benali^16^, Maurizio Berardino^17^, Luigi Beretta^9^, Morten Blaabjerg^18^, Peter Bragge^19^, Alexandra Brazinova^20^, Vibeke Brinck^21^, Joanne Brooker^22^, Camilla Brorsson^23^, Andras Buki^24^, Monika Bullinger^25^, Manuel Cabeleira^26^, Alessio Caccioppola^27^, Emiliana Calappi ^27^, Maria Rosa Calvi^9^, Peter Cameron^28^, Guillermo Carbayo Lozano^29^, Marco Carbonara^27^, Simona Cavallo^17^, Giorgio Chevallard^30^, Arturo Chieregato^30^, Giuseppe Citerio^31, 32^, Hans Clusmann^33^, Mark Coburn^34^, Jonathan Coles^35^, Jamie D. Cooper^36^, Marta Correia^37^, Amra Čović ^38^, Nicola Curry^39^, Endre Czeiter^40^, Marek Czosnyka^26^, Claire Dahyot‑Fizelier^41^, Paul Dark^42^, Helen Dawes^43^, Véronique De Keyser^44^, Vincent Degos^16^, Francesco Della Corte^45^, Hugo den Boogert^10^, Bart Depreitere^46^, Đula Đilvesi ^47^, Abhishek Dixit^48^, Emma Donoghue^22^, Jens Dreier^49^, Guy‑Loup  Dulière^50^, Ari Ercole^48^, Patrick Esser^43^, Erzsébet Ezer^51^, Martin  Fabricius^52^, Valery L. Feigin^53^, Kelly  Foks^54^, Shirin Frisvold^55^, Alex Furmanov^56^, Pablo Gagliardo^57^, Damien Galanaud^16^, Dashiell Gantner^28^, Guoyi Gao^58^, Pradeep George^59^, Alexandre Ghuysen^60^, Lelde Giga^61^, Ben Glocker^62^, Jagoš Golubovic^47^, Pedro A. Gomez ^63^, Johannes Gratz^64^, Benjamin Gravesteijn^65^, Francesca Grossi^45^, Russell L. Gruen^66^, Deepak Gupta^67^, Juanita A. Haagsma^65^, Iain Haitsma^68^, Raimund Helbok^69,70^, Eirik Helseth^71^, Lindsay Horton ^72^, Jilske Huijben^65^, Peter J. Hutchinson^73^, Bram Jacobs^74^, Stefan Jankowski^75^, Mike Jarrett^21^, Ji‑yao  Jiang^59^, Faye Johnson^76^, Kelly Jones^53^, Mladen Karan^47^, Angelos G. Kolias^73^, Erwin Kompanje^77^, Daniel Kondziella^52^, Evgenios Kornaropoulos^48^, Lars‑Owe Koskinen^78^, Noémi Kovács^79^, Ana Kowark^80^, Alfonso Lagares^63^, Linda Lanyon^59^, Steven Laureys^81^, Fiona Lecky^82, 83^, Didier Ledoux^81^, Rolf Lefering^84^, Valerie Legrand^85^, Aurelie Lejeune^86^, Leon Levi^87^, Roger Lightfoot^88^, Hester Lingsma^65^, Andrew I.R. Maas^44,89^, Ana M. Castaño‑León^63^, Marc Maegele^90^, Marek Majdan^20^, Alex Manara^91^, Geoffrey Manley^92^, Costanza Martino^93^, Hugues Maréchal^50^, Julia Mattern^94^, Catherine McMahon^95^, Béla Melegh^96^, David Menon^48^, Tomas Menovsky^44,89^, Ana Mikolic^65^, Benoit Misset^81^, Visakh Muraleedharan^59^, Lynnette Murray^28^, Ancuta Negru^97^, David Nelson^1^, Virginia Newcombe^48^, Daan Nieboer^65^, József Nyirádi^2^, Otesile Olubukola^82^, Matej Oresic^98^, Fabrizio Ortolano^27^, Aarno Palotie^99, 100, 101^, Paul M. Parizel^102^, Jean‑François Payen^103^, Natascha Perera^12^, Vincent Perlbarg^16^, Paolo Persona^104^, Wilco Peul^105^, Anna Piippo-Karjalainen^106^, Matti Pirinen^99^, Dana Pisica^65^, Horia Ples^97^, Suzanne Polinder^65^, Inigo Pomposo^29^, Jussi P. Posti ^107^, Louis Puybasset^108^, Andreea Radoi ^109^, Arminas Ragauskas^110^, Rahul Raj^106^, Malinka Rambadagalla^111^, Isabel Retel Helmrich^65^, Jonathan Rhodes^112^, Sylvia Richardson^113^, Sophie Richter^48^, Samuli Ripatti^99^, Saulius Rocka^110^, Cecilie Roe^114^, Olav Roise^115,116^, Jonathan Rosand^117^, Jeffrey V. Rosenfeld^118^, Christina Rosenlund^119^, Guy Rosenthal^56^, Rolf Rossaint^80^, Sandra Rossi^104^, Daniel Rueckert^62^ Martin Rusnák^120^, Juan Sahuquillo^109^, Oliver Sakowitz^94, 121^, Renan Sanchez‑Porras^121^, Janos Sandor^122^, Nadine Schäfer^84^, Silke Schmidt^123^, Herbert Schoechl^124^, Guus Schoonman^125^, Rico Frederik Schou^126^, Elisabeth Schwendenwein^6^, Charlie Sewalt^65^, Ranjit D. Singh^105^, Toril Skandsen^127, 128^ , Peter Smielewski^26^, Abayomi Sorinola^129^, Emmanuel Stamatakis^48^, Simon Stanworth^39^, Robert Stevens^130^, William Stewart^131^, Ewout W. Steyerberg^65,^ ^132, 133^, Nino Stocchetti^134^, Nina Sundström^135^, Riikka Takala^136^, Viktória Tamás^129^, Tomas Tamosuitis^137^, Mark Steven Taylor^20^, Aurore Thibaut^81^, Braden Te Ao^53^, Olli Tenovuo^107^, Alice Theadom^53^, Matt Thomas^91^, Dick Tibboel^138^, Marjolein Timmers^77^, Christos Tolias^139^, Tony Trapani^28^, Cristina Maria Tudora^97^, Andreas Unterberg^94^, Peter Vajkoczy ^140^, Shirley Vallance^28^, Egils Valeinis^61^, Zoltán Vámos^51^, Mathieu van der Jagt^141^, Gregory Van der Steen^44^, Joukje van der Naalt^74^, Jeroen T.J.M. van Dijck ^105^, Inge A. M. van Erp^105^, Thomas A. van Essen^105^, Wim Van Hecke^142^, Caroline van Heugten^143^, Ernest van Veen^65^, Thijs Vande Vyvere^144^, Roel P. J. van Wijk^105^, Alessia Vargiolu^32^, Emmanuel Vega^86^, Kimberley Velt^65^, Jan Verheyden^142^, Paul M. Vespa^145^, Anne Vik^127, 146^, Rimantas Vilcinis^137^, Victor Volovici^68^, Nicole von Steinbüchel^38^, Daphne Voormolen^65^, Petar Vulekovic^47^, Kevin K.W. Wang^147^, Daniel Whitehouse^48^, Eveline Wiegers^65^, Guy Williams^48^, Lindsay Wilson^72^, Stefan Winzeck^48^, Stefan Wolf^148^, Zhihui Yang^117^, Peter Ylén^149^, Alexander Younsi^94^, Marina Zeldovich^150^, Frederick A. Zeiler^48,151^, Veronika Zelinkova^20^, Agate Ziverte^61^ , Tommaso Zoerle^27^

^1^ Department of Physiology and Pharmacology, Section of Perioperative Medicine and Intensive Care, Karolinska Institutet, Stockholm, Sweden

^2^ János Szentágothai Research Centre, University of Pécs, Pécs, Hungary

^3^ Division of Clinical Neuroscience, Department of Physical Medicine and Rehabilitation, Oslo University Hospital and University of Oslo, Oslo, Norway

^4^ Department of Neurosurgery, University Hospital Northern Norway, Tromso, Norway

^5^ Department of Physical Medicine and Rehabilitation, University Hospital Northern Norway, Tromso, Norway

^6^ Trauma Surgery, Medical University Vienna, Vienna, Austria

^7^ Department of Anesthesiology & Intensive Care, University Hospital Nancy, Nancy, France

^8^ Raymond Poincare hospital, Assistance Publique – Hopitaux de Paris, Paris, France

^9^ Department of Anesthesiology & Intensive Care, S Raffaele University Hospital, Milan, Italy

^10^ Department of Neurosurgery, Radboud University Medical Center, Nijmegen, The Netherlands

^11^ Department of Neurosurgery, University of Szeged, Szeged, Hungary

^12^ International Projects Management, ARTTIC, Munchen, Germany

^13^ Department of Neurology, Neurological Intensive Care Unit, Medical University of Innsbruck, Innsbruck, Austria

^14^ Department of Neurosurgery & Anesthesia & intensive care medicine, Karolinska University Hospital, Stockholm, Sweden

^15^ NIHR Surgical Reconstruction and Microbiology Research Centre, Birmingham, UK

^16^ Anesthesie-Réanimation, Assistance Publique – Hopitaux de Paris, Paris, France

^17^ Department of Anesthesia & ICU, AOU Città della Salute e della Scienza di Torino - Orthopedic and Trauma Center, Torino, Italy

^18^ Department of Neurology, Odense University Hospital, Odense, Denmark

^19^ BehaviourWorks Australia, Monash Sustainability Institute, Monash University, Victoria, Australia

^20^ Department of Public Health, Faculty of Health Sciences and Social Work, Trnava University, Trnava, Slovakia

^21^ Quesgen Systems Inc., Burlingame, California, USA

^22^ Australian & New Zealand Intensive Care Research Centre, Department of Epidemiology and Preventive Medicine, School of Public Health and Preventive Medicine, Monash University, Melbourne, Australia

^23^ Department of Surgery and Perioperative Science, Umeå University, Umeå, Sweden

^24^ Department of Neurosurgery, Örebro University and University Hospital, Örebro, Sweden

^25^ Department of Medical Psychology, Universitätsklinikum Hamburg-Eppendorf, Hamburg, Germany

^26^ Brain Physics Lab, Division of Neurosurgery, Dept of Clinical Neurosciences, University of Cambridge, Addenbrooke’s Hospital, Cambridge, UK

^27^ Neuro ICU, Fondazione IRCCS Cà Granda Ospedale Maggiore Policlinico, Milan, Italy

^28^ ANZIC Research Centre, Monash University, Department of Epidemiology and Preventive Medicine, Melbourne, Victoria, Australia

^29^ Department of Neurosurgery, Hospital of Cruces, Bilbao, Spain

^30^ NeuroIntensive Care, Niguarda Hospital, Milan, Italy

^31^ School of Medicine and Surgery, Università Milano Bicocca, Milano, Italy

^32^ NeuroIntensive Care Unit, Department Neuroscience, IRCCS Fondazione San Gerardo dei Tintori, Monza, Italy

^33^Department of Neurosurgery, Medical Faculty RWTH Aachen University, Aachen, Germany

^34^ Department of Anesthesiology and Intensive Care Medicine, University Hospital Bonn, Bonn, Germany

^35^ Department of Anesthesia & Neurointensive Care, Cambridge University Hospital NHS Foundation Trust, Cambridge, UK

^36^ School of Public Health & PM, Monash University and The Alfred Hospital, Melbourne, Victoria, Australia

^37^ Radiology/MRI department, MRC Cognition and Brain Sciences Unit, Cambridge, UK

^38^ Institute of Medical Psychology and Medical Sociology, Universitätsmedizin Göttingen, Göttingen, Germany

^39^ Oxford University Hospitals NHS Trust, Oxford, UK

^40^ Department of Neurosurgery, Medical School, University of Pécs, Hungary and Neurotrauma Research Group, János Szentágothai Research Centre, University of Pécs, Hungary

^41^ Intensive Care Unit, CHU Poitiers, Potiers, France

^42^ University of Manchester NIHR Biomedical Research Centre, Critical Care Directorate,  Salford Royal Hospital NHS Foundation Trust, Salford, UK

^43^ Movement Science Group, Faculty of Health and Life Sciences, Oxford Brookes University, Oxford, UK

^44^ Department of Neurosurgery, Antwerp University Hospital, Edegem, Belgium

^45^ Department of Anesthesia & Intensive Care, Maggiore Della Carità Hospital, Novara, Italy

^46^ Department of Neurosurgery, University Hospitals Leuven, Leuven, Belgium

^47^ Department of Neurosurgery, Clinical centre of Vojvodina, Faculty of Medicine, University of Novi Sad, Novi Sad, Serbia

^48^ Division of Anaesthesia, University of Cambridge, Addenbrooke’s Hospital, Cambridge, UK

^49^ Center for Stroke Research Berlin, Charité – Universitätsmedizin Berlin, corporate member of Freie Universität Berlin, Humboldt-Universität zu Berlin, and Berlin Institute of Health, Berlin, Germany

^50^ Intensive Care Unit, CHR Citadelle, Liège, Belgium

^51^ Department of Anaesthesiology and Intensive Therapy, University of Pécs, Pécs, Hungary

^52^ Departments of Neurology, Clinical Neurophysiology and Neuroanesthesiology, Region Hovedstaden Rigshospitalet, Copenhagen, Denmark

^53^ National Institute for Stroke and Applied Neurosciences, Faculty of Health and Environmental Studies, Auckland University of Technology, Auckland, New Zealand

^54^ Department of Neurology, Erasmus MC, Rotterdam, the Netherlands

^55^ Department of Anesthesiology and Intensive care, University Hospital Northern Norway, Tromso, Norway

^56^ Department of Neurosurgery, Hadassah-hebrew University Medical center, Jerusalem, Israel

^57^ Fundación Instituto Valenciano de Neurorrehabilitación (FIVAN), Valencia, Spain

^58^ Department of Neurosurgery, Shanghai Renji hospital, Shanghai Jiaotong University/school of medicine, Shanghai, China

^59^ Karolinska Institutet, INCF International Neuroinformatics Coordinating Facility, Stockholm, Sweden

^60^ Emergency Department, CHU, Liège, Belgium

^61^ Neurosurgery clinic, Pauls Stradins Clinical University Hospital, Riga, Latvia

^62^ Department of Computing, Imperial College London, London, UK

^63^ Department of Neurosurgery, Hospital Universitario 12 de Octubre, Madrid, Spain

^64^ Department of Anesthesia, Critical Care and Pain Medicine, Medical University of Vienna, Austria

^65^ Department of Public Health, Erasmus Medical Center-University Medical Center, Rotterdam, The Netherlands

^66^ College of Health and Medicine, Australian National University, Canberra, Australia

^67^ Department of Neurosurgery, Neurosciences Centre & JPN Apex trauma centre, All India Institute of Medical Sciences, New Delhi-110029, India

^68^ Department of Neurosurgery, Erasmus MC, Rotterdam, the Netherlands

^69^ Department of Neurology, Kepler University Hospital, Johannes Kepler University Linz, Linz, Austria.

^70^ Clinical Research Institute for Neuroscience, Johannes Kepler University Linz, Linz, Austria

^71^ Department of Neurosurgery, Oslo University Hospital, Oslo, Norway

^72^ Division of Psychology, University of Stirling, Stirling, UK

^73^ Division of Neurosurgery, Department of Clinical Neurosciences, Addenbrooke’s Hospital & University of Cambridge, Cambridge, UK

^74^ Department of Neurology, University of Groningen, University Medical Center Groningen, Groningen, Netherlands

^75^ Neurointensive Care , Sheffield Teaching Hospitals NHS Foundation Trust, Sheffield, UK

^76^ Salford Royal Hospital NHS Foundation Trust Acute Research Delivery Team, Salford, UK

^77^ Department of Intensive Care and Department of Ethics and Philosophy of Medicine, Erasmus Medical Center, Rotterdam, The Netherlands

^78^ Department of Clinical Neuroscience, Neurosurgery, Umeå University, Umeå, Sweden

^79^ Hungarian Brain Research Program - Grant No. KTIA_13_NAP-A-II/8, University of Pécs, Pécs, Hungary

^80^ Department of Anaesthesiology, University Hospital of Aachen, Aachen, Germany

^81^ Cyclotron Research Center , University of Liège, Liège, Belgium

^82^ Centre for Urgent and Emergency Care Research (CURE), Health Services Research Section, School of Health and Related Research (ScHARR), University of Sheffield, Sheffield, UK

^83^ Emergency Department, Salford Royal Hospital, Salford UK

^84^ Institute of Research in Operative Medicine (IFOM), Witten/Herdecke University, Cologne, Germany

^85^ VP Global Project Management CNS, ICON, Paris, France

^86^ Department of Anesthesiology-Intensive Care, Lille University Hospital, Lille, France

^87^ Department of Neurosurgery, Rambam Medical Center, Haifa, Israel

^88^ Department of Anesthesiology & Intensive Care, University Hospitals Southhampton NHS Trust, Southhampton, UK

^89^ Department of Translational Neuroscience, Faculty of Medicine and Health Science, University of Antwerp, Antwerp, Belgium

^90^ Cologne-Merheim Medical Center (CMMC), Department of Traumatology, Orthopedic Surgery and Sportmedicine, Witten/Herdecke University, Cologne, Germany

^91^ Intensive Care Unit, Southmead Hospital, Bristol, Bristol, UK

^92^ Department of Neurological Surgery, University of California, San Francisco, California, USA

^93^ Department of Anesthesia & Intensive Care,M. Bufalini Hospital, Cesena, Italy

^94^ Department of Neurosurgery, University Hospital Heidelberg, Heidelberg, Germany

^95^ Department of Neurosurgery, The Walton centre NHS Foundation Trust, Liverpool, UK

^96^ Department of Medical Genetics, University of Pécs, Pécs, Hungary

^97^ Department of Neurosurgery, Emergency County Hospital Timisoara , Timisoara, Romania

^98^ School of Medical Sciences, Örebro University, Örebro, Sweden

^99^ Institute for Molecular Medicine Finland, University of Helsinki, Helsinki, Finland

^100^ Analytic and Translational Genetics Unit, Department of Medicine; Psychiatric & Neurodevelopmental Genetics Unit, Department of Psychiatry; Department of Neurology, Massachusetts General Hospital, Boston, MA, USA

^101^ Program in Medical and Population Genetics; The Stanley Center for Psychiatric Research, The Broad Institute of MIT and Harvard, Cambridge, MA, USA

^102^ Department of Radiology, University of Antwerp, Edegem, Belgium

^103^ Department of Anesthesiology & Intensive Care, University Hospital of Grenoble, Grenoble, France

^104^ Department of Anesthesia & Intensive Care, Azienda Ospedaliera Università di Padova, Padova, Italy

^105^ Dept. of Neurosurgery, Leiden University Medical Center, Leiden, The Netherlands and Dept. of Neurosurgery, Medical Center Haaglanden, The Hague, The Netherlands

^106^ Department of Neurosurgery, Helsinki University Central Hospital

^107^ Division of Clinical Neurosciences, Department of Neurosurgery and Turku Brain Injury Centre, Turku University Hospital and University of Turku, Turku, Finland

^108^ Department of Anesthesiology and Critical Care, Pitié -Salpêtrière Teaching Hospital, Assistance Publique, Hôpitaux de Paris and University Pierre et Marie Curie, Paris, France

^109^ Neurotraumatology and Neurosurgery Research Unit (UNINN), Vall d'Hebron Research Institute, Barcelona, Spain

^110^ Department of Neurosurgery, Kaunas University of technology and Vilnius University, Vilnius, Lithuania

^111^ Department of Neurosurgery, Rezekne Hospital, Latvia

^112^ Department of Anaesthesia, Critical Care & Pain Medicine NHS Lothian & University of Edinburg, Edinburgh, UK

^113^ Director, MRC Biostatistics Unit, Cambridge Institute of Public Health, Cambridge, UK

^114^ Department of Physical Medicine and Rehabilitation, Oslo University Hospital/University of Oslo, Oslo, Norway

^115^ Division of Orthopedics, Oslo University Hospital, Oslo, Norway

^116^ Institue of Clinical Medicine, Faculty of Medicine, University of Oslo, Oslo, Norway

^117^ Broad Institute, Cambridge MA Harvard Medical School, Boston MA, Massachusetts General Hospital, Boston MA, USA

^118^ National Trauma Research Institute, The Alfred Hospital, Monash University, Melbourne, Victoria, Australia

^119^ Department of Neurosurgery, Odense University Hospital, Odense, Denmark

^120^ International Neurotrauma Research Organisation, Vienna, Austria

^121^ Klinik für Neurochirurgie, Klinikum Ludwigsburg, Ludwigsburg, Germany

^122^ Division of Biostatistics and Epidemiology, Department of Preventive Medicine, University of Debrecen, Debrecen, Hungary

^123^ Department Health and Prevention, University Greifswald, Greifswald, Germany

^124^ Department of Anaesthesiology and Intensive Care, AUVA Trauma Hospital, Salzburg, Austria

^125^ Department of Neurology, Elisabeth-TweeSteden Ziekenhuis, Tilburg, the Netherlands

^126^ Department of Neuroanesthesia and Neurointensive Care, Odense University Hospital, Odense, Denmark

^127^ Department of Neuromedicine and Movement Science, Norwegian University of Science and Technology, NTNU, Trondheim, Norway

^128^ Department of Physical Medicine and Rehabilitation, St.Olavs Hospital, Trondheim University Hospital, Trondheim, Norway

^129^ Department of Neurosurgery, University of Pécs, Pécs, Hungary

^130^ Division of Neuroscience Critical Care, John Hopkins University School of Medicine, Baltimore, USA

^131^ Department of Neuropathology, Queen Elizabeth University Hospital and University of Glasgow, Glasgow, UK

^132^ Dept. of Department of Biomedical Data Sciences, Leiden University Medical Center, Leiden, The Netherlands

^133^ Julius Center for Health Sciences and Primary Care, University Medical Center Utrecht, Utrecht, The Netherlands

^134^ Department of Pathophysiology and Transplantation, Milan University, and Neuroscience ICU, Fondazione IRCCS Cà Granda Ospedale Maggiore Policlinico, Milano, Italy

^135^ Department of Radiation Sciences, Biomedical Engineering, Umeå University, Umeå, Sweden

^136^ Perioperative Services, Intensive Care Medicine and Pain Management, Turku University Hospital and University of Turku, Turku, Finland

^137^ Department of Neurosurgery, Kaunas University of Health Sciences, Kaunas, Lithuania

^138^ Intensive Care and Department of Pediatric Surgery, Erasmus Medical Center, Sophia Children’s Hospital, Rotterdam, The Netherlands

^139^ Department of Neurosurgery, Kings college London, London, UK

^140^ Neurologie, Neurochirurgie und Psychiatrie, Charité – Universitätsmedizin Berlin, Berlin, Germany

^141^ Department of Intensive Care Adults, Erasmus MC– University Medical Center Rotterdam, Rotterdam, the Netherlands

^142^ icoMetrix NV, Leuven, Belgium

^143^ Movement Science Group, Faculty of Health and Life Sciences, Oxford Brookes University, Oxford, UK

^144^ Radiology Department, Antwerp University Hospital and University of Antwerp, Edegem, Belgium

^145^ Director of Neurocritical Care, University of California, Los Angeles, USA

^146^ Department of Neurosurgery, St.Olavs Hospital, Trondheim University Hospital, Trondheim, Norway

^147^ Department of Emergency Medicine, University of Florida, Gainesville, Florida, USA

^148^ Department of Neurosurgery, Charité – Universitätsmedizin Berlin, corporate member of Freie Universität Berlin, Humboldt-Universität zu Berlin, and Berlin Institute of Health, Berlin, Germany

^149^ VTT Technical Research Centre, Tampere, Finland

^150^ Sigmund Freud University, Faculty of Psychotherapy Science, Vienna, Austria

^151^ Section of Neurosurgery, Department of Surgery, Rady Faculty of Health Sciences, University of Manitoba, Winnipeg, MB, Canada
